# Research on Roles of Mongolian Medical Warm Acupuncture in Inhibiting p38 MAPK Activation and Apoptosis of Nucleus Pulposus Cells

**DOI:** 10.1155/2018/6571320

**Published:** 2018-08-09

**Authors:** Sha Li, Lidao Bao, Lengge Si, Xiaohui Wang, Agula Bo

**Affiliations:** ^1^Department of Pharmacy, Affiliated Hospital of Inner Mongolia Medical University, Hohhot, Inner Mongolia 010059, China; ^2^Mongolian Medicine School, Inner Mongolia Medical University, Hohhot, Inner Mongolia 010110, China

## Abstract

**Background:**

Mongolian medical warm acupuncture has a desirable therapeutic effect on sciatica. Apoptosis of the nucleus pulposus cells is considered to play an important role in sciatica. Evidence has demonstrated that oxidative stress and its induced activation of the signaling pathways play important roles in sciatica. However, further research is expected to reveal whether Mongolian medical warm acupuncture can inhibit the apoptosis of nucleus pulposus cells and oxidative stress.

**Objective:**

To study the effect of the p38 MAPK pathway activated by the generated ROS on apoptosis and the expression of the genes related to the balance of the extracellular matrix metabolism during treatment of sciatica with Mongolian medical warm acupuncture.

**Method:**

The volume of the active oxygen generated in the nucleus pulposus cells was detected following intervention of Mongolian medical warm acupuncture. The p38 MAPK phosphorylation level was detected with Western blot. The genes are related to the metabolism of the nucleus pulposus extracellular matrix.

**Result:**

Mongolian medical warm acupuncture reduced the active oxygen within the nucleus pulposus cells and inhibited the activation of the p38 MAPK pathway (P=0.013). Meanwhile, it upregulated the gene expression of Type II collagen, aggrecan, Sox-9, and tissue matrix metalloproteinase reagent 1 (P-0.015; P=0.025; P=0.031; P=0.045) and downregulated the gene expression of matrix metalloproteinase 3 (P=0.015).

**Conclusion:**

Mongolian medical warm acupuncture may inhibit apoptosis of nucleus pulposus cells and activation of the extracellular matrix decomposition metabolism pathway and promote its anabolism. This process may rely on the oxidative stress matrix of the p38 MAPK pathway.

## 1. Introduction

Sciatica is a chronic process that influences some compositional, structural, and functional changes in intervertebral disc [1]. It is one of the major causes of lumbago. It can lead to loss of labor ability in many patients [2]. The intervertebral disc is the largest nonvascular tissue in the body. In addition to the end plate, the intervertebral disc mainly comprises two different categories of anatomical structures, i.e., internal nucleus pulposus and peripheral anulus fibrosus [3]. Apoptosis of the nucleus pulposus cells is considered to play an important role in sciatica [4]. Sciatica belongs to “white vein diseases”. In Mongolian medicine, it is believed that prevalence of Badagan and qi and blood operating disorders are the common pathological results arising from various pathogenic factors and the pathological basis of onset of pain. The three major factors lose their balance thus leading to ache, numbness, and muscular atrophy of lower limbs [5]. Treatment at the acupoints or joints aims to eliminate evils for supporting healthy energy, promote blood circulation for removing blood stasis, relax muscles and tendons and remove obstruction from meridians, relieve swelling and pain, and regulate the immune functions [6].

Previous research has indicated that excessive ROS (reactive oxygen species) can not only directly oxidize and damage DNA, protein, and lipid and but also activate a large number of stress-sensitive pathways [7] in the cells as signal molecules, such as p38 MAPK pathways, c-Jun N-terminal kinase (JNK) pathway [8], extracellular signal-regulated protein kinase (ERK) pathway, and nuclear factor-kappa B NF-B pathway [9]. Mongolian medical warm acupuncture relieves the pain because it negatively modulates the activation of stress pathways or due to the apoptosis induction of altered cells [10].

Although the causes of sciatica still remain unclear evidence has demonstrated that oxidative stress and its induced activation of the signaling pathways play important roles in sciatica [11–13]. The experiment is intended to study the effect of the p38 MAPK pathway activated by the generated ROS on apoptosis and the expression of the genes related to the balance of the extracellular matrix metabolism after the animal models are intervened by Mongolian medical warm acupuncture and the nucleus pulposus cells are extracted.

## 2. Material and Methods

### 2.1. Experimental Animals

Healthy male SPF Wistar rats aged 3 months weighing 200-250 g (Laboratory Animals Center of Inner Mongolia Medical University (IMMU), Hohhot, China, license number of experimental animals: SCXK Mongolian 2013-0004) were used. The rats were divided into 4 groups: (1) blank control group; (2) sciatica model group; (3) sciatica model + analgesic decoction group (Ibuprofen Sustained Release Capsule, Fenbid, H10900089, Sino-US Tianjin Shike, Tianjin, China, 50 mg/Kg/d, intragastric administration); (4) sciatica model + Mongolian medical warm acupuncture group. The specific establishment method was described as follows. They were fed a normal diet (Keao Xieli Feed Co. Ltd., Beijing, China) for 1 week. Food and water were supplied ad libitum. Rats were individually housed in cages and maintained at a constant temperature (23 ± 2°C) and humidity (55 ± 5%) and exposed to a 12-h light/dark cycle. The animals were cared for in accordance with the Guiding Principles in the Care and Use of Animals. All experiments were approved by the Institutional Animal Care and Use Committee of the Institute of Psychology of the IMMU.

### 2.2. Sciatica Rat Model

**Figure 1:**
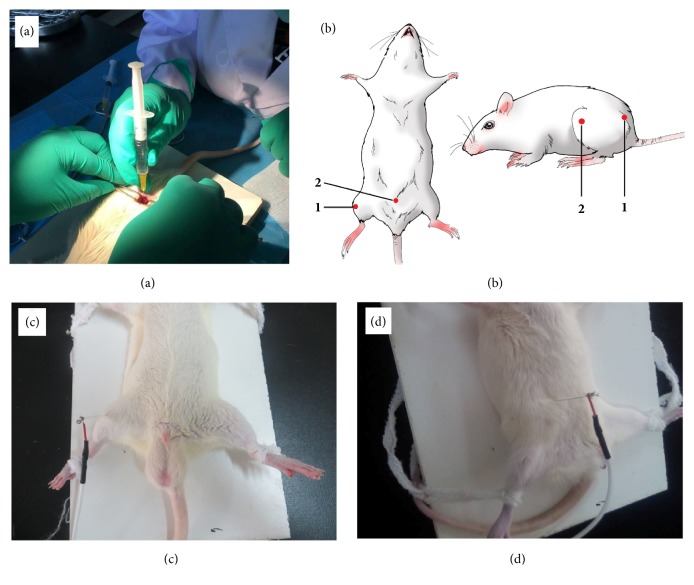
Schematic diagram of acupoints for treating sciatica with warm needle acupuncture. (a) Acupuncture and moxibustion. (b/c/d) Knee eye and hip acupoint anatomical positioning map.

After the rats were intraperitoneally injected with pentobarbital sodium for anesthetization, the skin of the left hind limbs was cut open. The muscle was bluntly separated to expose the sciatic nerve. It was loosely bundled with 4-0 catgut suture to establish sciatica rats with a chronic constriction injury (CCI) (Figure 1(a)). The rats were intramuscularly injected with penicillin for infection prevention. The paw withdrawal latency (PWL) of the rats was determined with the Model 336 radiant heat pain threshold detector (Shanghai Medical Apparatus and Instruments Factory, China). The PWL of the rats decreased significantly at 3 d after operation. Hyperalgesia was stable at 7 d. Warm needle intervention was performed from the 8th day after surgery [14].

### 2.3. Mongolian Medical Warm Acupuncture Therapy

Special needles (School of Mongolian Medical Pharmacy, Inner Mongolia Medical University, Patent No.: ZL201120058078.0) (0.5 mm in diameter, 3.0 cm in length) were obliquely inserted into the knee eye acupoint (midpoint of the line between the anterior superior spine and the most salient point of the greater trochanter of femur) and the hip acupoint (midpoint of the transverse texture of the lower hip). The Mongolian warm needling apparatus Model MLY-I (Patent Number: ZL201120058078.0) was connected (Figures 1(b)–1(d)). The rats were stimulated for 3 times of 15 min each time at an interval of 2 days at 40°C (current intensity: 100 mA). Rats do not need anesthesia during acupuncture.

### 2.4. Separation and Culture of the Nucleus Pulposus Cells of the Rats

After the third (day 7) acupuncture, the rats were euthanized by CO_2_ asphyxiation. The intervertebral disc tissue from L3 to L6 was taken. The adnexa in the rear area of the spine were cut out along the vertebral pedicle to take out the complete jelly-like nucleus pulposus tissue. The nucleus pulposus tissue was digested for 30 minutes with 4 mL 0.4% pronase (Roche, Indianapolis, IN) after being washed with Hanks' solution. Then, it was digested for 3 hours with 4 mL of 0.2% collagenase (Sigma, St. Louis, MO) and filtered with a 200-mesh screen. The supernatant was collected. The supernatant was centrifuged at 1000 rpm for 3 minutes. The supernatant was discarded. 5 mL of DMEM/F12 was added. It was placed in an incubator containing 5% C02 at 37°C. The solution was changed every two days following adherence of the primary cells. Cell viability was measured by MTT assay (method slightly).

### 2.5. Apoptosis Detection

The cultured cells were washed twice with the 0.1 mmol/L PBS solution (pH 7.4) and suspended in 190 *μ*l binding buffer. The cell concentration was adjusted to 1 × 10^6^/ml. 10 *μ*l of the fluorescein isothiocyanate- (FTIC-) marked connexin was added. Annexin and 10 *μ*l of 20 mg/L PI (BD Biosciences, San Jose, CA) were well mixed. It was incubated for 10 minutes at room temperature in the dark. It was washed once with the binding buffer and analyzed with the Coulter Elite (Beckman Coulter, Miami, FL) flow cytometer. The experiment detected apoptosis of the nucleus pulposus cells treated in various groups by means of Annexin-V-FITC/PI staining and the flow cytometry.

### 2.6. Detection of the Level of ROS (Reactive Oxygen Species)

The treated nucleus pulposus cells at the logarithmic phase were diluted and inoculated into a 6-well microplate (3×10^5^ cells per well). The cells were incubated for 3 min in the dark with DCFH-DA (BD Pharmingen, San Diego, CA, USA) with a final concentration of 10 *μ*mol/L. The cells were collected, washed twice with ice-precooled PBS, and detected for the level of ROS in the cells with the flow cytometer (Beckman Coulter, Miami, FL). The nucleus pulposus cells not treated with DCFH-DA served as the control; we detect the relative expression of fluorescence intensity.

### 2.7. Western Blot

The nucleus pulposus cells in the logarithmic growth phase were diluted and inoculated into a 6-well microplate. The cells in the control group and the treatment group were collected, respectively, lysed with RIPA, subjected to protein quantification, and detected with Western blot. SDS-polyacrylamide gel electrophoresis was performed. The protein sample was transferred to the nitrocellulose membrane, blocked, and incubated with the p38 MAPK antibody (dilution rate 1 : 1 000) (Miltenyi Biotec, Bergisch-Gladbach, Germany) and the GAPDH antibody (1 : 2 000) (Wuhan Boster Biological Engineering Co., Ltd., Wuhan, China), respectively, for 2 h at room temperature, incubated with the appropriate secondary antibody (Wuhan Boster Biological Engineering Co., Ltd., Wuhan, China) for 1 h following membrane washing, luminized, and developed with the chemiluminescence method.

### 2.8. RT-PCR

The primer synthesis was provided by (Sangon Biotech Bioengineering Co., Ltd., Shanghai, China). All primer designs use the Primer Premier 6.0 software (PREMIER Biosoft, CA, USA)—Type II collagen (COL2A1): L: agcctggtgatgatggtgaa; R: actctcacccttcacaccag; aggrecan: L: tccccaacagatgcttccat; R: gtacttgttccagccctcct; Sox-9: L: atgaagatgaccgacgagca; R: aacttgtcctcctcgctctc; MMP-3: L: cctggaaatgttttggccca; R: tcatcttgagacaggcggaa; TIMP-1: L: ccttctgcaattccgacctc; R: gtatccgcagacactctcca; GAPDH: L: gctcaacgtgtggtcatctc; R: acccttccacgatcccaaat. RT-PCR reaction reagent kit was purchased from Qiagen, Valencia, CA, USA. The RNA in the tissue was extracted routinely. 20 *μ*l of fluorescent RT-PCR reaction solution, 1 *μ*l of DNA polymerase, 0.35 *μ*l of reverse transcriptase, and 5 *μ*l of template RNA were well mixed and centrifuged at 6000 r/min for 10 s. Real-time fluorescent RT-PCR amplification procedures were as follows: 95°C 10 minutes, 95°C 10 seconds, 60°C 20 seconds, 40 cycles, and 72°C 15 seconds 4°C. The image was scanned for brightness with software Melanie III (GeneBio, Geneva, Switzerland).

### 2.9. Statistical Analysis

The data were expressed with mean ± standard error ($\overset{-}{x}$  ± SEM). Software SPSS 18.0 (SPSS Inc., Chicago, IL) was used for statistical analysis. For the intergroup differences the one-way analysis of variance and the Student-Newman-Keuls method were used. The nonmeasurement data were detected with Kruskal–Wallis test. The Nemenyi nonparametric analysis was used for further study. The difference was considered significant when P< 0.05.

## 3. Result

### 3.1. Viability of Nucleus Pulposus Cells in Various Treatment Groups at Different Points in Time

**Figure 2:**
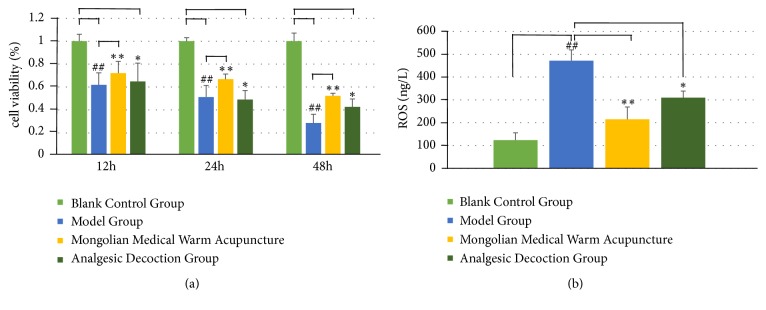
Survival rate of nucleus pulposus cells in different groups and effect on active oxygen in nucleus pulposus cells. (a) The viability of the cells in the Mongolian medical warm acupuncture group and the analgesic decoction intervention group increased. (b) It demonstrated that Mongolian medical warm acupuncture can reduce excessive ROS produced in the nucleus pulposus cells. This may be one of the causes of decreased apoptosis. # (##) indicates P<0.05 (P<0.01) when compared with the control group; *∗*(*∗∗*) indicates P<0.05 (P<0.01) when compared with the 25 mM glucose group.

To evaluate the effect of different treatment methods on the viability of the nucleus pulposus cells, the viability of the nucleus pulposus cells in the blank control group was set to 1. As shown in Figure 2(a), the viability of the nucleus pulposus cells in the model group decreased significantly when compared with that in the control group at each point in time (P=0.043). The viability of the cells in the Mongolian medical warm acupuncture group and the analgesic decoction intervention group increased when compared with that in the blank group (P=0.005, P=0.022). There was no significant difference between the Mongolian medical warm acupuncture group and the analgesic decoction group (P>0.05). The result showed that Mongolian medical warm acupuncture was able to significantly increase the viability of the nucleus pulposus cells. Its mechanism would be the focus in a further investigation.

### 3.2. Content of ROS in the Nucleus Pulposus Cells

The experiment used the fluorochrome DCF to detect ROS in the nucleus pulposus cells cultured under the condition of Mongolian medical warm acupuncture. The result indicated that the fluorescence intensity of the nucleus pulposus cells in the model group significantly increased (P=0.031) when compared with that in the control group (P=0.024). The content of ROS in the nucleus pulposus cells in the Mongolian medical warm acupuncture group decreased significantly when compared with that in the model group (P=0.006). It indicated that ROS in the cells in the Mongolian medical warm acupuncture group decreased when compared with that in the control group (Figure 2(b)). The content of ROS in the analgesic decoction decreased accordingly (P=0.018). It demonstrated that Mongolian medical warm acupuncture can reduce excessive ROS produced in the nucleus pulposus cells. This may be one of the causes of decreased apoptosis.

### 3.3. Apoptosis of Nucleus Pulposus Cells in Different Groups

**Figure 3:**
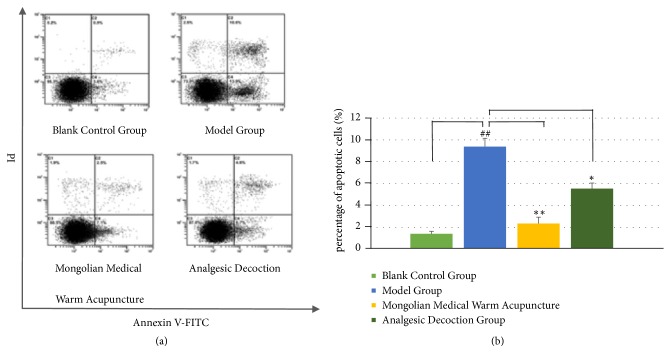
Apoptosis of nucleus pulposus cells in different groups. (a) The experiment detected apoptosis of the nucleus pulposus cells treated in various groups by means of Annexin-V-FITC/PI staining and the flow cytometry. (b) The result also indicated that Mongolian medical warm acupuncture was able to significantly decrease the apoptosis rate of the nucleus pulposus cells. # (##) indicates P<0.05 (P<0.01) when compared with the control group; *∗*(*∗∗*) indicates P<0.05 (P<0.01) when compared with the 25 mM glucose group.

To further define the effect of different treatment groups on cell survival, the result showed that the apoptotic rate of the nucleus pulposus cells in the model group increased significantly (P=0.012) and there was no significant difference in the apoptotic rate of the nucleus pulposus cells between the Mongolian medical warm acupuncture group and the control group (P>0.05). The apoptotic rate in the Mongolian medical warm acupuncture group significantly decreased when compared with that in the model group. The analgesic decoction was also able to improve apoptosis (P=0.023, P=0.032). The result also indicated that Mongolian medical warm acupuncture was able to significantly decrease the apoptosis rate of the nucleus pulposus cells. The result showed that Mongolian medical warm acupuncture was able to significantly affect the cell survival and apoptosis (Figure 3).

### 3.4. Detection of the Phosphorylation Level of p38 MAPK in the Nucleus Pulposus Cells

**Figure 4:**
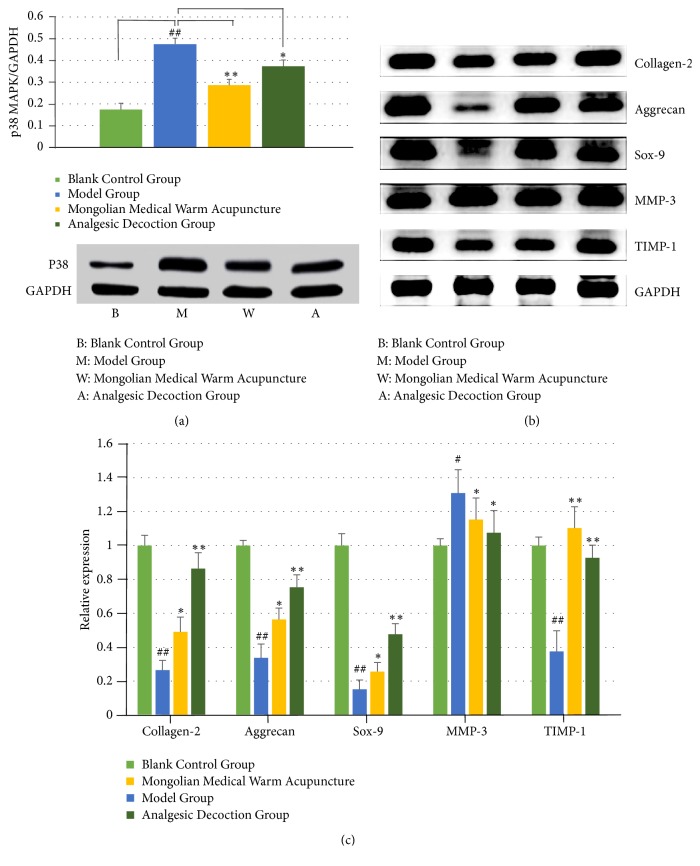
Phosphorylation level of p38 MAPK in the nucleus pulposus cells and effect of acupuncture on substrate metabolism of the rat intervertebral disc. (a) The phosphorylation level of p38 MAPK decreased following administration of analgesic decoction. (b/c) The expression of the Type II collagen, aggrecan, Sox-9, and TIMP-1 in the nucleus pulposus cells in the model group significantly decreased when compared with that in the control group. The expression of MMP-3 increased significantly when compared with that in the control group. # (##) indicates P<0.05 (P<0.01) when compared with the control group; *∗*(*∗∗*) indicates P<0.05 (P<0.01) when compared with the 25 mM glucose group.

The Western blot result showed that the phosphorylation level of p38 MAPK in the model group was significantly upregulated (P=0.013). It indicated that p38 MAPK phosphorylation was one of the mechanisms of sciatica. The application of Mongolian medical warm acupuncture was able to significantly decrease the phosphorylation level of p38 MAPK, indicating that Mongolian medical warm acupuncture was able to inhibit the p38 MAPK pathway. The phosphorylation level of p38 MAPK decreased following administration of analgesic decoction and there was no significant difference between the Mongolian medical warm acupuncture group and the analgesic decoction group, indicating that the efficacy of both therapies was nearly the same and they could inhibit the functions of p38 MAPK phosphorylation (Figure 4(a)).

### 3.5. Effect of Mongolian Medical Warm Acupuncture on Substrate Metabolism of the Rat Intervertebral Disc and the Genes Related to Apoptosis of Nucleus Pulposus Cells

The expression of Type II collagen, aggrecan, Sox-9, and TIMP-1 in the nucleus pulposus cells in the model group significantly decreased when compared with that in the control group. The expression of MMP-3 increased significantly when compared with that in the control group (P=0.033). The result showed that the sciatica model could inhibit the anabolism of the extracellular matrix and promote the catabolism of matrix. Meanwhile, the expression of Type II collagen, aggrecan, Sox-9, and TIMP-1 significantly increased when compared with that in the control group (P=0.015; P=0.025; P=0.031; P=0.045). The expression of MMP-3 decreased significantly when compared with that in the control group (P=0.015). The result showed that active oxygen and activation of the p38 MAPK pathway could influence the expression of the genes related to the nucleus pulposus matrix of the model rats (Figures 4(b) and 4(c)).

## 4. Discussion

A lot of research findings have shown that a significant decrease in the number of the cellular constituents in the degenerative intervertebral disc is one of the important reasons for extracellular substrate metabolism imbalance and decreased content [15, 16] while apoptosis is the leading cause of the decreased number of the cells in the intervertebral disc [17]. Wu D et al. have detected the apoptotic rate of the cells in the intervertebral disc of the patients with protrusion of intervertebral disc and discovered that the apoptotic rate is significantly higher than that in the healthy people [18]. Wang H et al. have discovered that apoptotic cells are present in the specimens of the nucleus pulposus and the inner fiber ring of the patients with protrusion of lumbar intervertebral disc and pointed out that apoptosis of the nucleus pulposus cells is achieved via the apoptosis pathway mediated by Fas, mitochondria, and the cytochrome C [19]. Inhibition of the pathway may defer cell apoptosis and slow down the progression of sciatica [20]. The experimental results have shown that the survival rate of the nucleus pulposus cells in the sciatica model group decreases while the cell apoptotic rate increases. The content of ROS generated in the nucleus pulposus cells in the Mongolian medical warm acupuncture group decreases. Excessive ROS leads to failure of the redox equilibrium in the cells and intracellular oxidative stress. A great deal of research on multiple different cell systems has demonstrated that ROS can induce apoptosis [21]. In addition, much research has discovered that antioxidants or antioxidant enzymes can inhibit apoptosis [22].

The experimental result has indicated that the model group and the subsequent apoptosis of nucleus pulposus cells induced by oxidative stress are mediated by activation of p38 MAPK. Mongolian medical warm acupuncture can significantly reduce the number of the apoptotic cells. In addition to direct apoptosis, some research findings have indicated that oxidative stress is associated with the accelerated aging of the cells in the degenerative intervertebral disc [23]. p38 MAPK is a kinase that attracts wide attention due to the fact that it can be activated by many endogenous or exogenous stress-induced stimulations [24, 25].

Mongolian medical warm acupuncture can inhibit activation of intracellular p38 MAPK. Both the increased ROS and activation of the p38 pathway can be inhibited by Mongolian medical warm acupuncture. In addition, the p38 MAPK pathway is associated with the extracellular substrate metabolism of the intervertebral disc. Moreover, it is worth noting that Mongolian medical warm acupuncture can regulate the expression of multiple mRNA transcripts related to balance of the extracellular substrate metabolism within the nucleus pulposus cells of the rats. Aggrecan and Type II collagen are the major constituents of the extracellular substrate of the nucleus pulposus cells [26].

In conclusion, Mongolian medical warm acupuncture can inhibit the apoptotic rate of the nucleus pulposus cells, inhibit extracellular substrate catabolism, and promote anabolism. This result can inhibit the activation pathway of p38 MAPK by inhibiting the increase of ROS.

## Data Availability

The data used to support the findings of this study are available from the corresponding author upon request.

## References

[1] Lassere M. N., Johnson K. R., Thom J., Pickard G., Smerdely P. (2018). Protocol of the randomised placebo controlled pilot trial of the management of acute sciatica (SCIATICA): a feasibility study. *BMJ Open*.

[2] Hofmann F., Stössel U., Michaelis M., Nübling M., Siegel A. (2002). Low back pain and lumbago-sciatica in nurses and a reference group of clerks: Results of a comparative prevalence study in Germany. *International Archives of Occupational and Environmental Health*.

[3] Sardaru D. P., Matei D., Zaharia-Kezdi D., Pendefunda L. (2018). Effects of biofeedback versus switch-triggered functional electrical stimulation on sciatica-related foot drop. *Journal of Back and Musculoskeletal Rehabilitation*.

[4] Tin S. S., Wiwanitkit V. (2014). Sciatica in the young. *Asian Spine Journal*.

[5] Fu L., Zhang H., Lu J., Zang H., Lou M., Wang G. (2015). Multilevel nonlinear mixed-effect crown ratio models for individual trees of Mongolian oak (quercus mongolica) in northeast China. *PLoS ONE*.

[6] Toyoda T., Shi L., Takasu S. (2016). Anti-inflammatory effects of capsaicin and piperine on helicobacter pylori-induced chronic gastritis in mongolian gerbils. *Helicobacter*.

[7] Tutoglu A., Boyaci A., Karababa İ. F. (2015). Psychological defensive profile of sciatica patients with neuropathic pain and its relationship to quality of life. *Zeitschrift für Rheumatologie*.

[8] Cass S. P. (2015). Piriformis syndrome: a cause of nondiscogenic sciatica. *Current Sports Medicine Reports*.

[9] Liu Z.-H., Miao G.-S., Wang J.-N., Yang C.-X., Fu Z.-J., Sun T. (2016). Resolvin d1 inhibits mechanical hypersensitivity in sciatica by modulating the expression of nuclear factor-*κ*B, phospho-extracellular signal-regulated kinase, and pro-and antiinflammatory cytokines in the spinal cord and dorsal root ganglion. *Anesthesiology*.

[10] Bo A., Si L., Wang Y., Xiu L., Wu R., Li Y. (2016). Clinical trial research on mongolian medical warm acupuncture in treating insomnia. *Evidence-Based Complementary and Alternative Medicine*.

[11] Hanqing T., Jianyu Z., Tianzi L. (2016). Effect of combined medicated thread moxibustion plus needle picking therapy of Zhuang nationality medicine on antioxidant levels in a rat model of sciatica. *Journal of Traditional Chinese Medicine*.

[12] Koda M., Mannoji C., Oikawa M. (2015). Herpes zoster sciatica mimicking lumbar canal stenosis: a case report. *BMC Research Notes*.

[13] Di Mattia F., Tejani S., Hall T. (2018). Bed rest for sciatica: a closer look at the evidence. *Journal of Orthopaedic and Sports Physical Therapy*.

[14] Ni H.-D., Yao M., Huang B. (2016). Glial activation in the periaqueductal gray promotes descending facilitation of neuropathic pain through the p38 MAPK signaling pathway. *Journal of Neuroscience Research*.

[15] Fouquet N., Bodin J., Chazelle E., Descatha A., Roquelaure Y. (2018). Use of multiple data sources for surveillance of work-related chronic low-back pain and disc-related sciatica in a french region. *Annals of Work Exposures and Health*.

[16] He B.-S., Li Y., Gui T. (2018). Preliminary clinical evaluation of acupuncture therapy in patients with postpartum sciatica. *Journal of Midwifery & Women’s Health*.

[17] Stynes S., Konstantinou K., Ogollah R., Hay E. M., Dunn K. M., Lammi M. J. (2018). Clinical diagnostic model for sciatica developed in primary care patients with low back-related leg pain. *PLoS ONE*.

[18] Di Wu, Zheng C., Wu J. (2017). Molecular biological effects of weightlessness and hypergravity on intervertebral disc degeneration. *Aerospace Medicine and Human Performance*.

[19] Wang H., Liu H., Zheng Z.-M. (2011). Role of death receptor, mitochondrial and endoplasmic reticulum pathways in different stages of degenerative human lumbar disc. *Apoptosis*.

[20] Dhoble S., Majumdar A. (2014). Detoxification of Abrus precatorius L. Seeds by Ayurvedic Shodhana process and anti-inflammatory potential of the detoxified extract. *Journal of Ayurveda and Integrative Medicine*.

[21] Wu H. Y., Huang C. H., Lin Y. H., Wang C. C., Jan T. R. (2018). Cannabidiol induced apoptosis in human monocytes through mitochondrial permeability transition pore-mediated ROS production. *Free Radical Biology & Medicine*.

[22] Radenkovic F., Holland O., Vanderlelie J. J., Perkins A. V. (2017). Selective inhibition of endogenous antioxidants with Auranofin causes mitochondrial oxidative stress which can be countered by selenium supplementation. *Biochemical Pharmacology*.

[23] Moses Z. B., Chi J. H. (2017). Genetic susceptibility for sciatica and lumbar disc herniation. *Neurosurgery*.

[24] Zhang Z., Ma W., Wang L. (2015). Activation of type 4 metabotropic glutamate receptor attenuates oxidative stress-induced death of neural stem cells with inhibition of JNK and p38 MAPK signaling. *Stem Cells and Development*.

[25] Goh G. Y. S., Winter J. J., Bhanshali F. (2018). NHR-49/HNF4 integrates regulation of fatty acid metabolism with a protective transcriptional response to oxidative stress and fasting. *Aging Cell*.

[26] Li P., Hou G., Zhang R. (2017). High-magnitude compression accelerates the premature senescence of nucleus pulposus cells via the p38 MAPK-ROS pathway. *Arthritis Research & Therapy*.

